# *RBFOX2* and alternative splicing in B-cell lymphoma

**DOI:** 10.1038/s41408-018-0114-3

**Published:** 2018-08-10

**Authors:** Hilmar Quentmeier, Claudia Pommerenke, Stephan H. Bernhart, Wilhelm G. Dirks, Vivien Hauer, Steve Hoffmann, Stefan Nagel, Reiner Siebert, Cord C. Uphoff, Margarete Zaborski, Hans G. Drexler, ICGC MMML-Seq Consortium

**Affiliations:** 1 Department of Human and Animal Cell Lines, Leibniz-Institute DSMZ-German Collection of Microorganisms and Cell Cultures, Braunschweig, Germany; 2 Transcriptome Bioinformatics Group - Interdisciplinary Centre for Bioinformatics, Leipzig University, Leipzig, Germany; 3 Computational Biology, Leibniz Institute on Aging – Fritz Lipmann Institute and Friedrich Schiller University Jena, Jena, Germany; 4 Institute of Human Genetics, Ulm University and Ulm University Medical Center, Ulm, Germany

*RBFOX2* is a master regulator of alternative splicing^1^. This RNA-binding protein (RBP) is expressed in the brain^2^, muscle^3^, and embryonic stem cells^4^. *RBFOX2* is required not only for the proper splicing of target RNAs, but also for cerebellar development^2^, myogenesis^3^, and for survival of human embryonic stem cells^4^.

Hitherto, not much is known about the expression and function of *RBFOX2* in hematopoetic tissues. In an early report, *RBFOX2* had been shown to be capable of promoting inclusion of exon16 in protein 4.1R^5^. This splicing event is important for erythropoiesis because it increases the affinity of 4.1R for target genes^5^. In a recent study, expression of *RBFOX2* was detected in the human T-cell line JURKAT and a functional antagonism of the RBPs *RBFOX2* and *CELF2* was demonstrated^6^. We wanted to describe the expression patterns of *RBFOX2* in hematopoetic malignancies, to discover target genes and to unravel the consequence of *RBFOX2* repression for target gene splicing and isoform expression.

Expression array and Western blot analysis showed that human B non-Hodgkin lymphoma (B-NHL) cell lines are *RBFOX2* negative or positive (Supplement 1A). To find the potential targets of the splice factor *RBFOX2*, we compared the expression of individual exons in *RBFOX2*-negative and *RBFOX2*-positive cell lines. This approach relied on the assumption that the differential expression of this RBP would provoke changes in the expression of individual exons and would thereby allow identification of target genes. Supplement 1B shortlists these genes ordered by statistical significance. Expression of the individual exons and joining sequences of *MALT1* is shown as heatmap in Fig. 1a. The full-length *MALT1* variant was associated with *RBFOX2* expression (Fig. 1a).

**Fig. 1:**
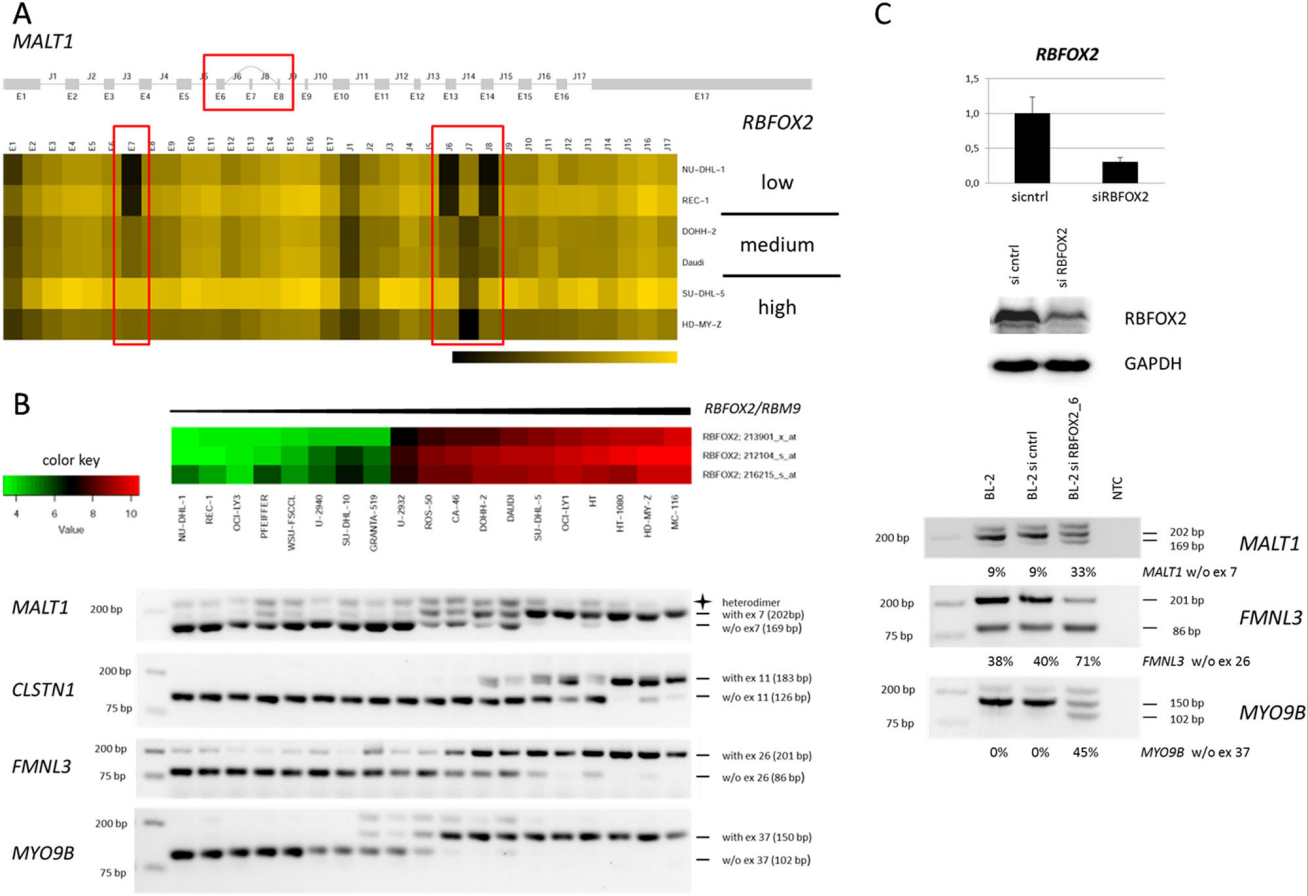
*RBFOX2* and *RBFOX2* targets in B-NHL cell lines. **a** Heatmap of RNAseq data showing expression of individual *MALT1* exons and corresponding joining sequences (red boxes: exon 7 and exon 7 joining sequences). Expression of full-length *MALT1* correlates with expression of *RBFOX2*. **b** Expression array analysis (upper) and RT-PCR analysis (lower) revealed that increasing expression of *RBFOX2* was paralleled by the full-length isoforms of *MALT1, CLSTN1, FMNL3*, and *MYO9B*. Exon numbering refers to the following sequences: *MALT1* (NM_006785.3), *CLSTN1* (NM_001009566), *FMNL3* (ENST00000550488.5), *MYO9B* (NM_001130065). **c** Transfection with siRNA oligonucleotides efficiently downregulated expression of *RBFOX2* mRNA (upper) and protein (medium). Repression of *RBFOX2* resulted in an increase of the short isoforms of *MALT1, FMNL3* and *MYO9B* (lower)

Results of splice variant analysis with a larger panel of cell lines revealed a striking association between expression of *RBFOX2* and expression of the full-length forms of all four candidate target genes, *MALT1, CLSTN1, FMNL3*, and *MYO9B* (Fig. 1b). The short variants were expressed in *RBFOX2-*negative cell lines only (Fig. 1b). Two of these potential *RBFOX2* target genes (*CLSTN1* and *FMNL3*) had already been described in the context of *RBFOX2*-mediated splicing^7^. The *RBFOX2* target sequence “UGCAUG” was present in all introns following the retained exons, indicating that high *RBFOX2* levels might be the cause of the full-length forms in the *RBFOX2* positive cell lines. Supporting the notion that *RBFOX2* was important for splicing of these genes was also the finding that *RBFOX2* was the sole gene that was significantly overexpressed in cell lines expressing full-length *MALT1* when compared to cell lines expressing *MALT1* without exon 7 (Supplement 1C).

We performed knockdown experiments to test whether *RBFOX2* was responsible for retaining *MALT1* exon 7, *FMNL3* exon 26, and *MYO9B* exon 37. siRNAs reduced expression of *RBFOX2* in *RBFOX2*-positive cell lines BL-2, SU-DHL-5, and HT by more than 50% (Fig. 1c, Supplement 1D). Repression of *RBFOX2* induced the shorter isoforms of *MALT1* (w/o exon 7), *MYO9B* (w/o exon 37), and *FMNL3* (w/o exon 26) (Fig. 1c, Supplement 1D). The long form of *CLSTN1*, the fourth gene tested here, was not or only marginally expressed in BL-2, SU-DHL-5, and HT cells, explaining why we could not observe an increase of the short isoform of this gene after *RBFOX2* knockdown (data not shown). In sum, our data showed that isoforms of *MALT1, MYO9B*, and *FMNL3* in B-NHL cell lines are controlled by *RBFOX2*: the full-length RNAs were expressed when *RBFOX2* was high, the short variants prevailed when *RBFOX2* was repressed.

*RBFOX2* is part of a mesenchymal splicing network^7^. The gene is also essential for the viability of human embryonic stem cells and for normal cerebellar development of mice^2,4^. Thus, *RBFOX2* appears to have different functions in cells of different origin. To check whether *RBFOX2* induced splicing of *MALT1, CLSTN1, FMNL3*, and *MYO9B* in hematopoetic cells other than B-cells, we tested *RBFOX2* expression and expression of the splice variants of the putative *RBFOX2* targets in T- and myeloid cell lines.

Neither T-cell lines nor myeloid cell lines reached the *RBFOX2* mRNA expression level of the B-cell line HT. Nevertheless, the T- and myeloid cell lines with highest *RBFOX2* mRNA levels also expressed the protein (Supplement 1A). T-cell lines with high *RBFOX2* levels expressed the large isoform of *MALT1*. However, an apparent universal dependence of *RBFOX2* and the large isoforms of *MALT1, CLSTN1, FMNL3*, and *MYO9B* comparable to that in B-cell lines was neither found in T- nor in myeloid cell lines. This does not necessarily mean that *RBFOX2* is functionless in cells of the T-lymphoid and myeloid lineages. *RBFOX2* is expressed in cell lines of both entities, and the full-length *MALT1* isoform is expressed in *RBFOX2-*positive T-cells. The other three genes (*CLSTN1, FMNL3*, and *MYO9B*) appear to be targets of *RBFOX2* in the B-lineage only. These results suggest that tissue-specific factors might contribute to the splicing process mediated by *RBFOX2*.

We limited our further studies to B-NHL, because we had identified the *RBFOX2* target genes in B-NHL cell lines. As shown in cell lines, also primary tumor cells of patients with diffuse large B-cell lymphoma (DLBCL) show differential *RBFOX2* gene expression (Fig. 1b upper, Supplement 1E). We analyzed RNAseq data from patients with different forms of B-NHL (ICGC MMML-Seq consortium) to find out whether primary tumor cells exhibited the same correlation between *RBFOX2* expression and the *RBFOX2* target gene isoforms as detected in B-NHL cell lines. We checked samples from patients with DLBCL (*n* = 78), Burkitt lymphoma (BL) (*n* = 21), follicular lymphoma (FL) (*n* = 87), and FL-DLBCL (*n* = 15). Germinal center (GC) B-cells (*n* = 5) and naive B-cells (*n* = 5) were included as controls.

*RBFOX2* expression and *MALT1* exon 7 inclusion were positively correlated in BL, FL, activated B-cell (ABC), and GC DLBCL (*p* < 0.05) (Table 1). In contrast, no such correlation was found for healthy controls, DLBCL (type III) and FL-DLBCL (Table 1). Supporting the notion that *RBFOX2* regulates splicing in all four proposed *RBFOX2* target genes (*MALT1* exon 7, *CLSTN1* exon 11, *FMNL3* exon 26, and *MYO9B* exon 37), we found a statistically significant positive correlation between expression of *RBFOX2* and inclusion of target exons in FL, BL, and in at least one subtype of DLBCL (Table 1). The data had been normalized against target gene expression levels to avoid a potential bias through target gene expression levels.

**Table 1:** Correlation between expression of *RBFOX2* and inclusion of exons in *RBFOX2* target genes

	*MALT1* exon 7		*CLSTN1* exon 11		*FMNL3* exon 26		*MYO9B* exon 37	
	Correlation	*p*-value	Correlation	*p*-value	Correlation	*p*-value	Correlation	*p*-value
DLBCL ABC (*n* = 26)	0.496	**0.009**	0.450	**0.023**	0.139	0.496	0.338	0.09
DLBCL GCB (*n* = 37)	0.591	**0.0001**	0.147	0.383	0.558	**0.0004**	0.561	**0.0003**
DLBCL type III (*n* = 15)	0.125	0.675	0.385	0.156	1	0	0.307	0.265
All DLBCL (*n* = 78)	0.522	**1.2** **x** **10**^**−6**^	0.303	**0.007**	0.300	**0.008**	0.566	**1.1** **x** **10**^**−7**^
BL (solid ped BL) (*n* = 21)	0.703	**0.0005**	0.669	**0.001**	0.484	**0.027**	0.672	**0.001**
FL (*n* = 87)	0.321	**0.003**	0.437	**2.3** **x** **10**^**−5**^	0.227	**0.034**	0.397	**0.0001**
FL-DLBCL (*n* = 15)	0.464	0.083	0.029	0.923	0.503	0.058	0.435	0.106
GC B cells, control (*n* = 5)	−0.9	0.083	0.2	0.783	0.3	0.683	−0.2	0.783
Naive B cells, control (*n* = 5)	0.6	0.083	-0.354	0.783	-0.8	0.683	−0.2	0.783

RNASeq data from lymphoma and control, mapped to hg38 with segemehl 2.0; data were normalized against target

gene expression. Bold: statistically significant. Normalization: transformation to target gene expression levels

*RBFOX2* is a member of the *RBFOX* family of RBP, also including *RBFOX1* and *RBFOX3*. All three proteins recognize the same sequence (UGCAUG) in regulated exons or in flanking introns^8^. To analyze whether *RBFOX1* and *RBFOX3* might also contribute to the splicing of our four target genes, we tested for correlation between expression of these *RBFOX* family members and inclusion of exons in target genes. We did not find a statistically significant correlation between *RBFOX1* or *RBFOX3* expression and inclusion of exons in *CLSTN1* and *FMNL3* (Supplement 1F). *MALT1* and *MYO9B* showed this correlation only in selected tumor variants, but not in BL, ABC DLBCL, or GC DLBCL, when the latter two were analyzed as individual lymphoma entities (Supplement 1F). Thus, *RBFOX2* was the only *RBFOX* family member whose expression was positively correlated with the full-length isoforms of the target genes (*MALT1, CLSTN1, FMNL3*, and *MYO9B*) in BL, FL, and DLBCL.

These data suggest that *RBFOX2* is a regulator of splicing in B-NHL. This notion is based on (i) the positive correlation between *RBFOX2* expression and expression of the full-length variants of the putative *RBFOX2* target genes in B-NHL cell lines and in primary B-NHL samples, and (ii) results of knockdown experiments demonstrating that *RBFOX2* is responsible for inclusion of exons in *MALT1* and other target genes.

*MALT1* appears to be of special interest as it encodes a protease that activates the IKK complex^9^. In lymphocytes, MALT1 cleaves RelB, which also leads to the activation of NFkB^10^. Both *MALT1* isoforms (with and w/o exon 7) are expressed in T-lymphocytes and expression of the individual variants has consequences for T-cell receptor triggered signal transduction^11^. As part of the *CARMA1–BCL10–MALT1* complex, *MALT1* is also a central regulator of the B-cell receptor (BCR) / NFkB pathway. ABC-type DLBCL cells rely on the constitutive activation of this pathway to block apoptosis^12^. Recurrent mutations in *CD79A/B*, *CARD11*, and other BCR/NFkB pathway genes have been described^13^. Like *Brutons Tyrosine Kinase*, upstream to *MALT1* in the BCR/NFkB pathway, also *MALT1* is a potential target for precision therapy^14^. Future studies shall elucidate whether the two *MALT1* isoforms display different capacities to activate NFkB in B-NHL, which might be of importance for the clinical application of MALT1 inhibitors.

In summary, (i) *RBFOX2* is expressed in hematopoetic cell lines of different origin; (ii) expression of *RBFOX2* correlates with isoforms of potential target genes in B-NHL cell lines and in primary B-NHL cells; and (iii) knockdown experiments suggest that *RBFOX2*—directly or indirectly—contributes to the splicing of target genes including *MALT1*, a protease in the BCR/NFkB pathway.

## Electronic supplementary material

Supplemental Methods

Supplemental Figure
