# Supplementary material for: RBFOX2 and alternative splicing in B-cell lymphoma
Source: Blood Cancer J. 2018 Aug 10;8(8):77. doi: 10.1038/s41408-018-0114-3 (PMC6086906; doi:10.1038/s41408-018-0114-3)
Supplement: Supplementary file 1 — Supplemental Methods [file 41408_2018_114_MOESM1_ESM.docx]

**Supplementary Methods**

***Human cell lines***

All cell lines are held by the DSMZ cell lines bank (<http://www.dsmz.de>) and were cultivated as described previously.^1^

***Patient cohort***

The ICGC MMML-Seq study has been approved by the ethics committees of the Medical Faculty of the University of Kiel (A150/10) and of the recruiting centers including Ulm University (329/11). The ICGC MMML-Seq cohort consists of pretreatment tumor tissue and corresponding germline material obtained with informed consent of the respective patients and/or their legal guardians, in the case of minors.

***Expression array analysis***

Expression array datasets (Affymetrix GeneChip HG-U133 Plus 2.0) obtained for cell lines and DLBCL patients (GSE53786)^2^ were organized using commonly used spreadsheet programs. Data processing and analysis (probe summarisation, RMA-background correction and quantile normalization of spot intensities) were performed via R(Bioconductor using limma and affy packages).^3-5^

***RNAseq analysis***

Public RNAseq datasets of immortalized cell lines were obtained from CCLE. CCLE data were retrieved via genetorrent from CGHub (https://cghub.ucsc.edu/software/downloads/GeneTorrent/3.8.7). Acquired bam files (70-110 Mio reads) were converted to fastq files via bedtools2. Reads were trimmed via fastq-mcf (ea-utils 1.04.807) and aligned by STAR to the Gencode Homo sapiens genome (v26) and converted/sorted via samtools.^6,7^ For differential usage of transcript exons and splice junctions expression analysis, reads were counted via QoRTs at first and then data was processed and analysed in the R/Bioconductor environment loading DESeq2 and JunctionSeq packages.^8-10^ Library size correction, data normalization, calculation of dispersions, testing for differentially used exons and splice junctions and correction for multiple testing were set as default in JunctionSeq. On basis of gene expression analysis via microarray analysis, five *RBFOX2* positive and five *RBFOX2* negative cell lines were selected to achieve a sufficient sample size for splice variant testing. Heatmaps for differentially used splicing variants were produced via SGSeq.^11,12^

Lymphoma RNASeq data was analysed as part of the german MMMLSeq ICGC consortium and has been published and uploaded to the european genome phenome archive (<https://www.ebi.ac.uk/ega/>) as part of several publications^13-17^. We mapped non strand-sensitive RNASeq data to the human reference genome hg38 on the SUPERMUC computer using segemehl^18^ version 0.3.0beta (version available on demand). Segemehl output contains a list of all spliced reads, which were collected and counted. Exon inclusion was measured using the number of reads spanning the splice site corresponding to the longest isoform (inclusion splice events) divided by the number of reads excluding the respective exon (exlusion splice events), see Supplement 1B.

As exon inclusion correlated with gene expression, we normalized the exon inclusion by dividing by the respective genes fragments per million (fpm) values. Gene expression was measured using featureCounts version 1.5.2^19^ and the gencode 27 genome annotation, featureCounts was used with the following parameters: -p, -M, --fraction, -O, -exon and 5 threads. Correlation between gene expression and exon inclusion was computed using R’s cor.test, applying spearman’s method to minimize the impact of outliers.

***Detection of splice variants by RT-PCT and quantification of RBFOX2 mRNA***

RNA was prepared using the RNeasy Mini kit (Qiagen, Hilden, Germany). Reverse transcription was performed using the SuperScript II reverse transcriptase kit (Invitrogen, Karlsruhe, Germany). Primers and RT-PCR conditions for detection of splice variants of *CLSTN1, FMNL3, MALT1* and *MYO9B* are shown in Supplement 1G.

Quantitative PCR (qRT-PCR) was performed on a 7500 Applied Biosystems (Darmstadt, Germany) real-time PCR system using the manufacturer`s protocol. A TaqMan probe (Applied Biosystems) was used to quantify human *RBFOX2* (Hs00204814_m1) expression levels with *TATA box binding protein* (*TBP*) as endogenous control.

***Knock-down experiments***

Transfection with siRNA oligonucleotides was performed as described.^20^ *RBFOX2* specific siRNA oligonucleotides and AllStars negative control siRNA were obtained from Qiagen (Hilden, Germany). siRNAs (80 pmol) were transfected into 1x10^6^ cells using the EPI-2500 impulse generator (Fischer, Heidelberg, Germany) at 350 V for 10 ms.

***Western blot analysis***

Abs against RBFOX2 were obtained from Atlas Antibodies/Biozol (Eching, Germany) and from LSBio/Biozol (Eching, Germany), Ab against GAPDH was purchased from Abcam (Camridge, UK). Western blot samples were prepared as described.^21^ Bands on nitrocellulose membranes were visualized with the biotin/streptavidin-horseradish peroxidase system (GE Healthcare; Little Chalfont, UK) in combination with the “Renaissance Western Blot Chemoluminescence Reagent” (Perkin Elmer; Waltham, MA, USA).

**References to Supplementary Methods**

1 Drexler HG. Guide to leukemia-lymphoma cell lines. 2^nd^ edition, Braunschweig, 2010.

2 Scott DW, Wright GW, Williams PM, Lih CJ, Walsh W, Jaffe ES *et al*. Determining cell-of origin subtypes of diffuse large B-cell lymphoma using gene expression in formalin-fixed paraffin-embedded tissue. *Blood* 2014; **123:** 1214-1217.

3 Ritchie ME, Phipson B, Wu D, Hu Y, Law CW, Shi W, *et al*. Limma powers differential expression analyses for RNA-sequencing and microarray studies*. Nucleic Acids Res* 2015; **43:** e47.

4 Gautier L, Cope L, Bolstad BM, Irizarry RA. Affy-analysis of Affymetrix GeneChip data at the probe level. *Bioinformatics* 2004; **20:** 307-315.

5 Dai M, Wang P, Boyd AD, Kostov G, Athey B, Jones EG *et al*. Evolving gene/transcript definitions significantly alert the interpretation of GeneChip data. *Nucleic Acids Res* 2005; **33:** e175.

6 Dobin A, Davis CA, Schlesinger F, Drenkow J, Zaleski C, Jha S *et al*. STAR: ultrafast universal RNA-seq aligner. *Bioinformatics* 2013; **29:**15-21.

7 Li H, Handsaker B, Wysoker A, Fennell T, Ruan J, Homer N *et al*. The sequence alignment/map (SAM) format and SAMtools. *Bioinformatics* 2009; **25:** 2078-2079.

8 Hartley SW, Mullikin JC. Qorts: a comprehensive toolset for quality control and data processing of rna-seq experiments. *BMC Bioinformatics* 2015; **16:** 224.

9 www.bioconductor.org.

10 Hartley SW, Mullikin JC. Detection and visualization of differential exon and splice junction usage in RNA-seq data with JunctionSeq. *arXiv* 2015; 1512.06038.

11 Anders S, Huber W. Differential expression analysis for sequence count data. *Genome Biol* 2010; **11:** R106.

12 Goldstein LD, Cao Y, Pau G, Lawrence M, Wu TD, Seshagiri S *et al*. Prediction and quantification of splice events from RNA-seq data. *PLos One* 2016; **11:** e0156132.

13 Hezaveh K, Kloetgen A, Bernhart SH, Mahapatra KD, Lenze D, Richter J *et al*. Alterations of microRNA and microRNA-regulated messenger RNA expression in germinal center B-cell lymphomas determined by integrative sequencing analysis. *Haematologica* 2016; **101:** 1380–1389.

14 Kretzmer H, Bernhart SH, Wang W, Haake A, Weniger MA, Bergmann AK *et al*. DNA-methylome analysis in Burkitt and follicular lymphomas identifies differentially methylated regions linked to somatic mutation and transcriptional control. *Nat Genet* 2015; **47:** 1316–1325.

15 Richter J, Schlesner M, Hoffmann S, Kreuz M, Leich E, Burkhardt B *et al*. Recurrent mutation of the ID3 gene in Burkitt lymphoma identified by integrated genome, exome and transcriptome sequencing. *Nat Genet* 2012; **44:** 1316–1320.

16 Lopez, C. *et al*. Genomic and transcriptomic changes complement each other in the pathogenesis of sporadic Burkitt lymphoma in children. in preparation

17 Hübschmann, D. *et al*. Mutational mechanisms shaping the coding and non-coding genome of B-cell lymphomas. in preparation

18 Hoffmann S, Otto C, Doose G, Tanzer A, Langenberger D, Christ S, *et al*. A multi-split mapping algorithm for circular RNA, splicing, trans-splicing, and fusion detection", *G*[*enome Biol*](http://www.ploscompbiol.org/article/info%3Adoi%2F10.1371%2Fjournal.pcbi.1000502) 2014; **15:** R34.

19 Liao Y, Smyth GK, Shi W. [featureCounts: an efficient general purpose program for assigning sequence reads to genomic features.](http://www.ncbi.nlm.nih.gov/pubmed/24227677) *Bioinformatics* 2014; 30: 923-30.

20 Nagel S, Ehrentraut S, Tomasch J, Lienenklaus S, Schneider B, Geffers R, *et al*. Transcriptional activation of prostate specific homeobox gene NKX3-1 in subsets of T-cell lymphoblastic leukemia (T-ALL). *PLoS One* 2012; **7:** e40747.

21 Quentmeier H, Schneider B, Röhrs S, Romani J, Zaborski M, MacLeod RAF, *et al*. SET-NUP214 fusion in acute myeloid leukemia- and T-cell acute lymphoblastic leukemia-derived cell lines. *J Hematol Oncol* 2009; **2:** 3.

**Full list of members of the ICGC MMML-Seq**

*Coordination (C1):* Reiner Siebert^1,2^, Susanne Wagner^2^, Andrea Haake^2^, Julia Richter^2,3^, Gesine Richter^2^

*Data Center (C2):* Roland Eils^4,5^, Chris Lawerenz^4^, Jürgen Eils^4^, Jules Kerssemakers^4^, Christina Jaeger-Schmidt^4^, Ingrid Scholz^4^

*Clinical Centers (WP1):* Anke K. Bergmann^2, 6^*,* Christoph Borst^7^, Birgit Burkhardt^8,9^, Alexander Claviez^6^, Martin Dreyling^10^, Sonja Eberth^11^, Hermann Einsele^12^, Norbert Frickhofen^13^, Siegfried Haas^7^, Martin-Leo Hansmann^14^, Dennis Karsch^15^, Michael Kneba^15^, Jasmin Lisfeld^9^, Luisa Mantovani-Löffler^16^, Marius Rohde^9^, German Ott^17^, Christina Stadler^11^, Peter Staib^18^, Stephan Stilgenbauer^19^, Lorenz Trümper^11^ , Thorsten Zenz^20^

*Normal Cells (WPN):* Martin-Leo Hansmann^14^, Dieter Kube^11^, Ralf Küppers^21^, Marc Weniger^21^

*Pathology and Analyte Preparation (WP2-3):* Michael Hummel^22^, Wolfram Klapper^3^, Ulrike Kostezka^23^, Dido Lenze^22^, Peter Möller^24^, Andreas Rosenwald^25^, German Ott^17^, Monika Szczepanowski^3^

*Sequencing and genomics (WP4-7):* Ole Ammerpohl^1,2^, Sietse M. Aukema^2,3^, Vera Binder^26^, Arndt Borkhardt^26^, Andrea Haake^2^, Jessica I. Hoell^26^; Ellen Leich^25^, Peter Lichter^27^, Cristina López^1,2^, Inga Nagel^2^, Jordan Pischimariov^25^, Bernhard Radlwimmer^27^, Julia Richter^2,3^, Philip Rosenstiel^28^, Andreas Rosenwald^25^, Markus Schilhabel^28^, Stefan Schreiber^29^, Inga Vater^2^, Rabea Wagener^1,2^, Reiner Siebert^1,2^

*Bioinformatics (WP8-9):* Stephan H. Bernhart^30-32,^ Hans Binder^30,31^, Benedikt Brors^33^, Gero Doose^30-32^, Roland Eils^4,5^, Steve Hoffmann^30-32^, Lydia Hopp^30^, Daniel Hübschmann^4,5,34^, Kortine Kleinheinz^4,5^, Helene Kretzmer^30-32^, Markus Kreuz^35^, Jan Korbel^36^, David Langenberger^30-32^, Markus Loeffler^35^, Maciej Rosolowski^35^, Matthias Schlesner^4,37^ , Peter F. Stadler^30-32,38-40^, Stephanie Sungalee^36^

^1^Institute of Human Genetics, University of Ulm and University Hospital of Ulm, Ulm, Germany

^2^Institute of Human Genetics, Christian-Albrechts-University, Kiel, Germany;

^3^Hematopathology Section, Institute of Pathology, Christian-Albrechts-University, Kiel, Germany;

^4^Division of Theoretical Bioinformatics (B080), German Cancer Research Center (DKFZ), Heidelberg, Germany;

^5^Department for Bioinformatics and Functional Genomics, Institute of Pharmacy and Molecular Biotechnology and Bioquant, University of Heidelberg, Heidelberg, Germany;

^6^Department of Pediatrics, University Hospital Schleswig-Holstein, Campus Kiel, Kiel, Germany;

^7^Department of Internal Medicine/Hematology, Friedrich-Ebert-Hospital, Neumünster;

^8^University Hospital Muenster - Pediatric Hematology and Oncology, Muenster Germany;

^9^University Hospital Giessen, Pediatric Hematology and Oncology, Giessen, Germany;

^10^Department of Medicine III - Campus Grosshadern, University Hospital Munich, Munich, Germany;

^11^Department of Hematology and Oncology, Georg-August-University of Göttingen, Göttingen, Germany;

^12^University Hospital Würzburg, Department of Medicine and Poliklinik II, University of Würzburg, Würzburg;

^13^Department of Medicine III, Hematology and Oncology, Dr. Horst-Schmidt-Kliniken of Wiesbaden, Wiesbaden;

^14^Senckenberg Institute of Pathology, University of Frankfurt Medical School, Frankfurt am Main, Germany

^15^Department of Internal Medicine II: Hematology and Oncology, University Medical Centre, Campus Kiel, Kiel;

^16^Hospital of Internal Medicine II, Hematology and Oncology, St-Georg Hospital Leipzig, Leipzig, Germany;

^17^Department of Pathology, Robert-Bosch-Hospital, Stuttgart, Germany;

^18^Clinic for Hematology and Oncology, St.-Antonius-Hospital, Eschweiler;

^19^Department for Internal Medicine III, University of Ulm and University Hospital of Ulm, Ulm, Germany

^20^National Centre for Tumor Disease, Heidelberg, Germany;

^21^Institute of Cell Biology (Cancer Research), University of Duisburg-Essen, Duisburg-Essen, Medical School, Essen, Germany;

^22^Institute of Pathology, Charité – University Medicine Berlin, Berlin, Germany;

^23^Comprehensive Cancer Center Ulm (CCCU), University Hospital Ulm, Ulm, Germany;

^24^Institute of Pathology, University of Ulm and University Hospital of Ulm, Ulm;

^25^Institute of Pathology, University of Wurzburg, Germany;

^26^Department of Pediatric Oncology, Hematology and Clinical Immunology, Heinrich-Heine-University, Düsseldorf, Germany;

^27^German Cancer Research Center (DKFZ), Division of Molecular Genetics, Heidelberg, 69120, Germany;

^28^Institute of Clinical Molecular Biology, Christian-Albrechts-University, Kiel, Germany;

^29^Department of General Internal Medicine, University Kiel, Kiel, Germany;

^30^Interdisciplinary Center for Bioinformatics, University of Leipzig, Leipzig, Germany;

^31^Bioinformatics Group, Department of Computer, University of Leipzig, Leipzig, Germany; ^32^Transcriptome Bioinformatics, LIFE Research Center for Civilization Diseases, University of Leipzig, Leipzig, Germany;

^33^Division of Applied Bioinformatics (G200), German Cancer Research Center (DKFZ), Heidelberg, Germany

^34^Department of Pediatric Immunology, Hematology and Oncology, University Hospital, Heidelberg, Germany

^35^Institute for Medical Informatics Statistics and Epidemiology, University of Leipzig, Leipzig, Germany;

^36^EMBL Heidelberg, Genome Biology, Heidelberg, Germany;

^37^Bioinformatics and Omics Data Analytics (B240), German Cancer Research Center (DKFZ), Heidelberg, Germany;

^38^RNomics Group, Fraunhofer Institute for Cell Therapy and Immunology IZI, Leipzig, Germany

^39^Santa Fe Institute, Santa Fe, New Mexico, United States of America

^40^Max-Planck-Institute for Mathematics in Sciences, Leipzig, Germany.
