# Supplementary material for: RBFOX2 and alternative splicing in B-cell lymphoma
Source: Blood Cancer J. 2018 Aug 10;8(8):77. doi: 10.1038/s41408-018-0114-3 (PMC6086906; doi:10.1038/s41408-018-0114-3)
Supplement: Supplementary file 2 — Supplemental Figure [file 41408_2018_114_MOESM2_ESM.pdf]

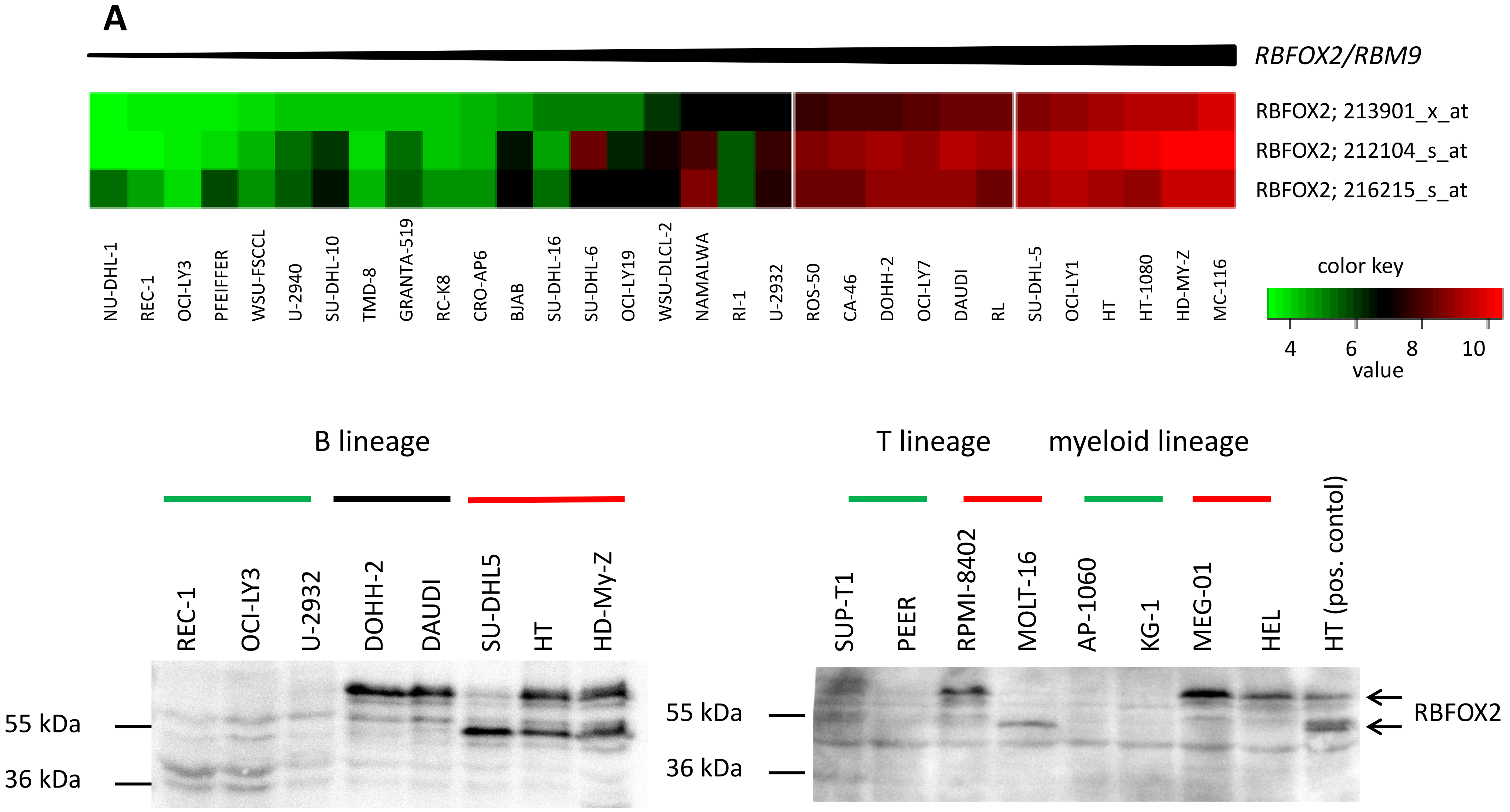

Supplement 1A – *RBFOX2* expression in B-NHL cell lines. Upper: Heatmap of expression array data showing expression of *RBFOX2* in human B-NHL cell lines. Cell lines HT-1080 (fibrosarcoma) and HD-MY-Z (a myelomonocytic cell line) were included as positive controls. Lower left: Western blot analysis confirmed expression of the RBFOX2 protein in *RBFOX2* mRNA positive cell lines. Lower right: Expression of the RBFOX2 protein in *RBFOX2* mRNA positive T- and myeloid cell lines. Green bar: *RBFOX2* mRNA negative; black bar: medium; red bar: high. The two sizes observed in Western blots may result from glycosylation of the RBFOX2 protein or they may represent two different *RBFOX2* isoforms.

B

Top 10 alternatively spliced genes in *RBFOX2* high vs *RBFOX2* low cell lines.

gene	gene location (hg38)			p-value	spliced exon	reference	exon location (hg38)	inclusion splice event	exclusion splice event
TRPM4	chr 19	49,157,740	49,211,836	1 e-131	n.a.	n.a.	n.a.	n.a.	n.a.
MYO9B	chr 19	17,075,780	17,214,537	5.05 e-110	exon 37	NM_001130065	19:17,210,333 - 17,210,380	19:17,209,709-17,210,332	19:17,209,709-17,210,714
FMNL3	chr 12	49,636,498	49,708,165	2.99 e-75	exon 26	ENST00000550488.5	12:49,646,639 - 49,646,753	12:49,645,903-49,646,638	12:49,645,903-49,646,885
ARNT	chr 1	150,809,704	150,876,768	1 e-40	n.a.	n.a.	n.a.	n.a.	n.a.
MALT1	chr 18	58,671,385	58,754,477	9.38 e-27	exon 7	NM_006785.3	18:58,710,921 - 58,710,953	18:58,710,072-58,710,920	18:58,710,072-58,714,082
KYAT1	chr 9	128,832,941	128,882,494	6.07 e-25	n.a.	n.a.	n.a.	n.a.	n.a.
S100A6	chr 1	153,534,598	153,536,244	8.83 e-25	n.a.	n.a.	n.a.	n.a.	n.a.
MYO10	chr 5	16,661,913	16,936,276	8.83 e-25	n.a.	n.a.	n.a.	n.a.	n.a.
CLSTN1	chr 1	9,729,025	9,824,526	1.96 e-25	exon 11	NM_001009566	1:9,736,040 - 9,735,885	1:9,736,042-9,737,497	1:9,736,042-9,741,093
LRP8	chr 1	53,242,363	53,328,070	1.71 e-23	n.a.	n.a.	n.a.	n.a.	n.a.

RNAseq analysis was performed for five *RBFOX2* positive cell lines (HD-MY-Z, HT, HT-1080, MC-116, SU-DHL-5) vs. five *RBFOX2* negative cell lines (GRANTA-519, NU-DHL-1, PFEIFFER, REC-1, SU-DHL-10). Genes with differentially expressed exons are listed according to statistical significance (p-values). Bold: Gene tested as potential RBFOX2 targets; n.a. not analyzed. The same exons (*MYO9B, FMNL3, MALT1,CLSTN1)* were spliced in primary B-NHL cells (see Table 1).

C

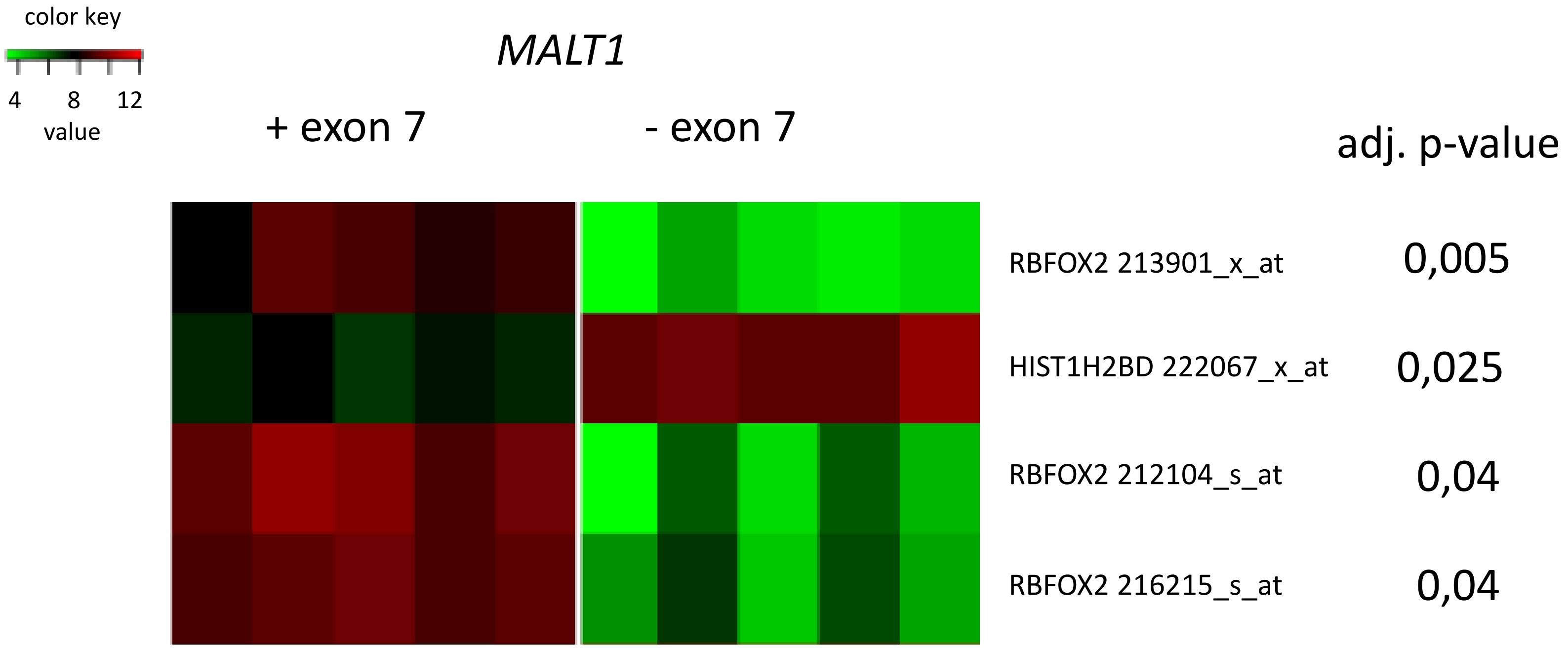

Supplement 1C – Over- and underexpressed genes in cell lines expressing different isoforms of *MALT1*. Comparison of expression array data revealed that only two genes were significantly differently expressed in B-NHL cell lines with the full-length isoform of *MALT1* vs. cell lines that expressed the isoform without exon 7. *RBFOX2* was overexpressed, *HIST1H2BD* was repressed in cell lines with full-length *MALT1*.

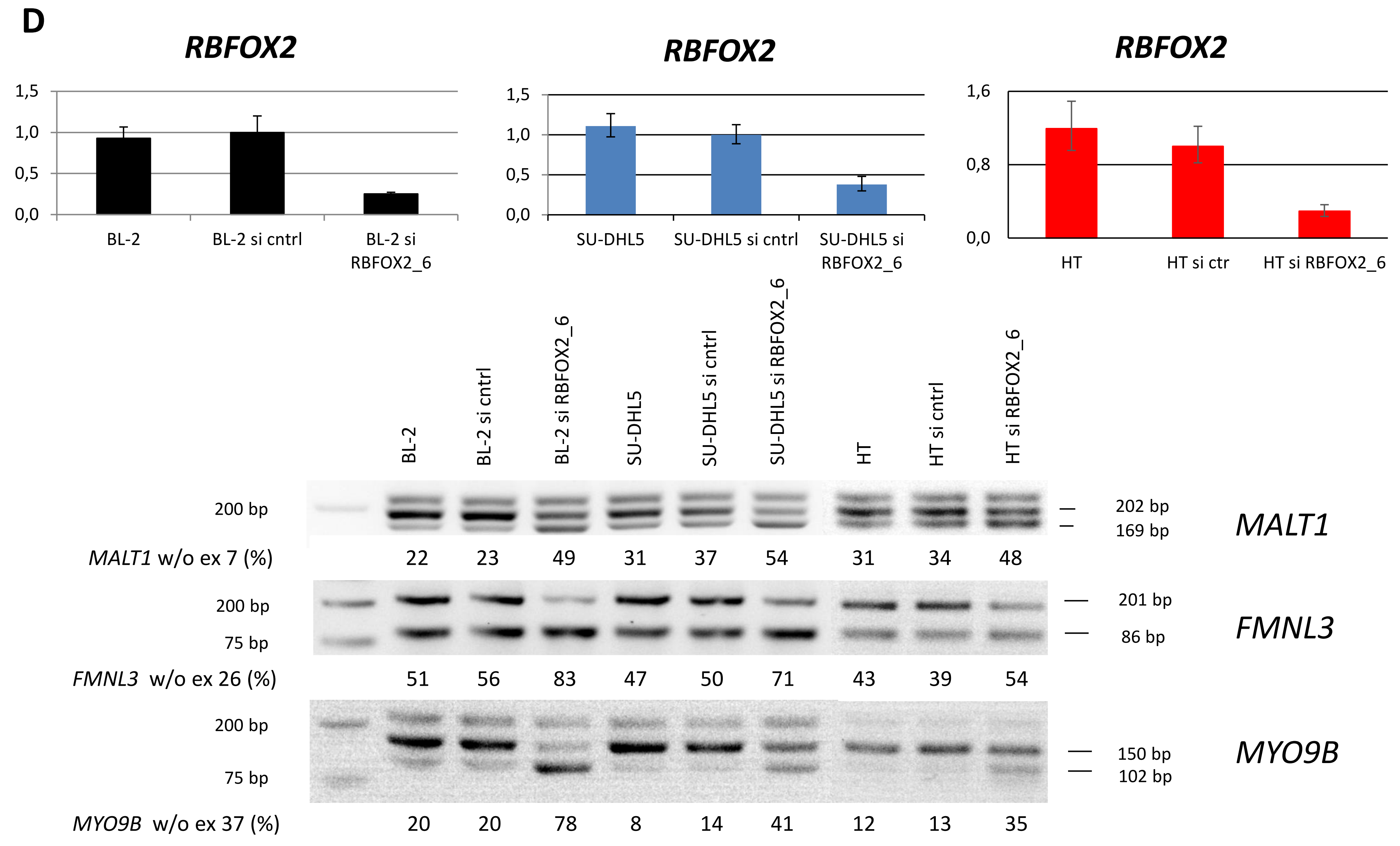

Supplement 1D – Knockdown of *RBFOX2* in B-NHL cell lines. Upper: Transfection with siRNA oligonucleotides efficiently downregulated expression of *RBFOX2* mRNA in cell lines BL-2, SU-DHL5 and HT. Lower: Repression of *RBFOX2* resulted in an increase of the short isoforms of *MALT1, FMNL3* and *MYO9B*.

E

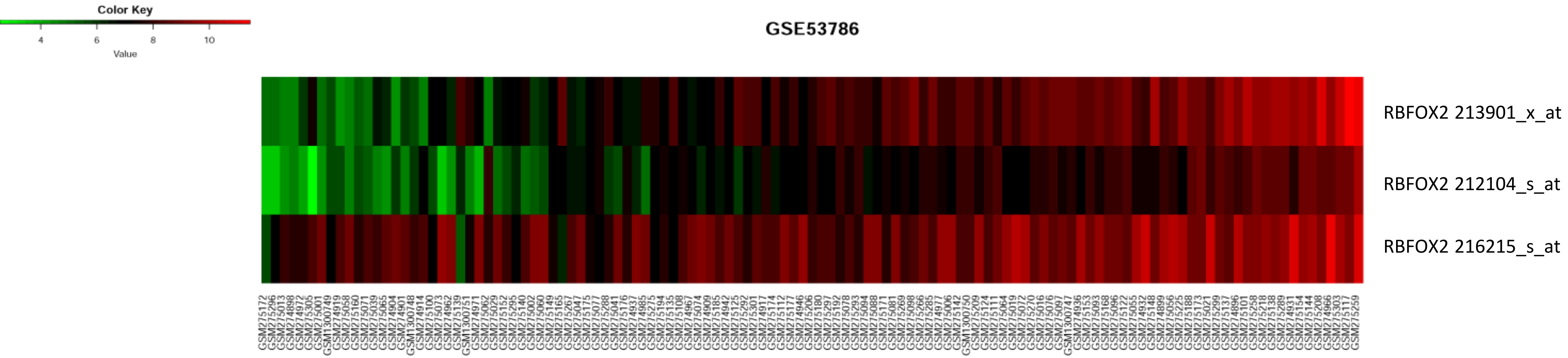

Supplement 1E – Expression of *RBFOX2* in DLBCL. Data of expression array analysis demonstrate that primary DLBCL lymphoma cells can be *RBFOX2*-negative or -positive (GSE53786).

F

Normalized target exon inclusion vs *RBFOX1* or *RBFOX3* .

	MALT1 exon 7<br>p-value	*CLSTN1* exon 11<br>p-value	*FMNL3* exon 26<br>p-value	*MYO9B* exon 37<br>p-value
DLBCL ABC (n=26)	> 0.05	> 0.05	> 0.05	> 0.05
DLBCL GCB (n=37)	> 0.05	> 0.05	> 0.05	> 0.05
DLBCL type III (n=15)	> 0.05	> 0.05	> 0.05	> 0.05
all DLBCL (n=78)	**0.012 / 0.007**	> 0.05	> 0.05	**0.003 /** > 0.05
BL (solid ped BL) (n=21)	> 0.05	> 0.05	> 0.05	> 0.05
FL (n=87)	> 0.05	> 0.05	> 0.05	**0.019** / > 0.05
FL-DLBCL (n=15)	**0.013** / > 0.05	> 0.05	> 0.05	> 0.05
GC B cells, control (n=5)	> 0.05	> 0.05	> 0.05	> 0.05
naive B cells, control (n=5)	> 0.05	> 0.05	> 0.05	> 0.05

RNASeq data from lymphoma and control, mapped to hg38 with segemehl 2.0. Bold: statistically significant. Black: valid for RBFOX1 and RBFOX3; red: RBFOX1-related values; blue: RBFOX3-related values.

G

Sequence of primers for the recognition of splice variants.

gene		RT-PCR primer
CLSTN1 exon 10	forward	5´- GAT AGA AAC TCA GCT CGT GGT - 3´
CSLTN1 exon 12	reverse	5´- ATC ACC TTC TTA TCC GCG AGT - 3´
FMNL3 exon 25	forward	5´- TGG TAC CAT CGA GGA CAT CAT - 3´
FMNL3 exon 27	reverse	5´- CAC TGG GCG GTG CAG CA - 3´
MALT1 exon 6	forward	5´- TGG AAC ACC AAG GAA CCT ACT -3´
MALT1 exon 10	reverse	5´- AGG GCA ACC TTG TCC TTC G - 3´
MYO9B exon 36	forward	5´- CAG AGA GTA TCG CCT TCC G - 3´
MYO9B exon 38	reverse	5´- CCA GCA GCA CCT CCA GC - 3´

PCR conditions: 59°C, 35 cycles.
